# A Nanoporous Polymer Modified with Hexafluoroisopropanol to Detect Dimethyl Methylphosphonate

**DOI:** 10.3390/nano14010089

**Published:** 2023-12-28

**Authors:** Xuming Wang, Xin Li, Qiang Wu, Yubin Yuan, Weihua Liu, Chuanyu Han, Xiaoli Wang

**Affiliations:** 1 Department of Microelectronics, Xi’an Jiaotong University, Xi’an 710049, China; 2 School of Physics, Xi’an Jiaotong University, Xi’an 710049, China

**Keywords:** surface acoustic wave, hexafluoroisopropanol, gas sensor, dimethyl methylphosphonate

## Abstract

The increasing threat of nerve agents has prompted the need for gas sensors with fast response, high sensitivity, and good stability. In this work, the hexafluoroisopropanol functional group was modified on a porous aromatic framework material, which served as a sensitive material for detecting dimethyl methylphosphonate. A nerve agent sensor was made by coating sensitive materials on a surface acoustic wave device. Lots of pores in sensitive materials effectively increase the specific surface area and provide channels for diffusion of gas molecules. The introduction of hexafluoroisopropanols enables the sensor to specifically adsorb dimethyl methylphosphonate and improves the selectivity of the sensor. As a result, the developed gas sensor was able to detect dimethyl methylphosphonate at 0.8 ppm with response/recovery times of 29.8/43.8 s, and the detection limit of the gas sensor is about 0.11 ppm. The effects of temperature and humidity on the sensor were studied. The results show that the baseline of the sensor has a linear relationship with temperature and humidity, and the temperature and humidity have a significant effect on the response of the sensor. Furthermore, a device for real-time detection of nerve agent is reported. This work provides a new strategy for developing a gas sensor for detecting nerve agents.

## 1. Introduction

Nerve agents are a category of volatile organic compounds containing phosphorus, including tabon, sarin, soman, and VX. Nerve agents can permeate the skin and respiratory system, covalently binding to acetylcholinesterase and resulting in the accumulation of acetylcholine within nerve cells. This, in turn, causes neurological disorders and ultimately leads to the death of humans and organisms [1]. Due to their high lethality and stealthy nature, nerve agents are easily used in terrorist attacks and pose a major threat to public safety. A nerve agent sensor is an effective early warning device and has attracted the attention of various countries. The sensor used for early warning must meet the requirements of fast response, high sensitivity, and anti-interference. In general, the sensors need to detect the leaked nerve agent within a minute, without interference from common organic volatiles such as ethanol and acetone. In addition to this, the size and cost of nerve agent sensors are also important factors to consider.

Common methods for detecting nerve agents include resistive gas sensors [2,3], metal oxide gas sensors [4], surface acoustic wave (SAW) gas sensors [5], infrared gas sensors [6], ion mobility spectrometry [7], gas phase mass spectrometry [8], etc. While metal oxide gas sensors offer high sensitivity, low cost, and compact size, they exhibit poor selectivity. In contrast, infrared gas sensors, ion mobility spectrometry, and gas phase mass spectrometry demonstrate exceptional selectivity, but their high cost and large size constrain their application scope. SAW gas sensors have the advantages of high sensitivity, small size, and low cost, making them highly promising in various applications [9]. A typical SAW gas sensor consists of an interdigital electrode, a piezoelectric substrate and a gas-sensitive film which is applied along the surface acoustic wave propagation path. The target gas absorption in the sensitive film alters the surface acoustic wave speed and accordingly modulates the amplitude or phase of the sensor output signal. The sensitive material significantly influences the sensitivity, response speed, and selectivity of the SAW gas sensor, and is therefore often a key part of SAW gas sensors [10,11].

In the development of nerve agent sensors, dimethyl methylphosphonate (DMMP) is usually used as the nerve agent simulator for the sake of safety. The molecular structure of DMMP is similar to that of nerve agents, as both molecules possess P=O functional groups [12]. The oxygen atom within the P=O group demonstrates strong electronegativity, which is easily attracted by positively charged functional groups. The hydrogen of the hydroxyl group in hexafluoroisopropanol (HFIP) carries a positive charge and is capable of forming hydrogen bonds with the oxygen in the P=O functional group [13]. By modifying the HFIP group on the sensitive film, DMMP and nerve agent can be effectively adsorbed. HFIP groups have successfully been modified to polydimethylsiloxane [14], graphene [15], carbon nanotubes (CNTs) [16], and metal oxides [17] and have shown excellent properties. However, for HFIP-modified polymers, the glass conversion temperature (T_g_) is an important parameter that affects the sensitivity and response speed of the sensor. When the sensor is below the T_g_, the fluidity of the polymer becomes poor, which hinders the adsorption and diffusion of DMMP. Accordingly, the sensitivity and response speed of the sensor are reduced. The T_g_ is an inherent property of polymer materials, which is usually difficult to change. For CNTs and metal oxide materials, because of the extremely high surface area, the sensitive material is very easy to agglomerate when preparing the sensor. In order to prevent agglomeration, surface modification of nanomaterials after preparation into three-dimensional frameworks is a common method, which further increases the processing complexity of the sensor.

Porous materials are a promising candidate because of their designability, flexible processes, and wide variety [18]. Compared with the above materials, solid porous materials can overcome the negative effects of T_g_ and also improve the sensitivity of the sensor. Porous materials, such as metal–organic framework materials [19], porous carbon [20], molecular sieves [21], and porous polymers offer flexibility for gas sensors. Porous polymers can detect gases including ammonia, hydrogen sulfide, hydrogen chloride, nitro explosives, etc. [22,23,24]. Nevertheless, porous polymers used for nerve agent detection have rarely been reported.

In this work, a porous HFIP-modified polymer was prepared via ultraviolet-induced polymerization utilizing divinylbenzene (DVB) and 2-Allylhexafluoroisopropanol (BTHB). The molecules of DVB were polymerized to form a stable spatial skeleton structure, which is also known as the porous aromatic framework (PAF). The molecular structure of BTHB contains vinyl, which allows it to be firmly modified on the polymer in the form of covalent bonds. The material contains a large number of pores, which increases the specific surface area, exposes more HFIP groups, and facilitates the molecular diffusion of DMMP. This enables the sensor to perform with excellent sensitivity and selectivity and fast response speed. The SAW gas sensor can detect 0.8 ppm of DMMP within 1 min, and corresponding real-time detection equipment is implemented. The effects of temperature and humidity on SAW gas sensors were explored, and the results show that the baseline of the sensor is linear with temperature and humidity. As humidity increases, the sensitivity of the sensor decreases. With the increase of temperature, the sensitivity and response time of the sensor decrease. The interaction between common volatile organic compounds, HFIP, and the benzene ring was calculated by first principles. The results show that the hydrogen bond between HFIPs is a key factor limiting the sensitivity of the sensor. This work provides an effective solution for the development of nerve agent sensors.

## 2. Materials and Methods

### 2.1. Design of SAW Device

A SAW delay-line structure with 200 nm thick aluminum interdigital transducers (IDTs) was fabricated on a 128° YX LiNbO_3_ wafer using a standard ultraviolet photolithography process. The delay-line length between the IDTs was 2.56 mm. The operating frequency of the designed SAW device was 152 MHz, and the SAW velocity on the 128° YX LiNbO_3_ wafer was 3890 m/s. Thus, the corresponding wavelength λ was 25.6 μm. The apertures of the two IDTs were 100 λ and their lengths were 130 λ and 40 λ, respectively. The prepared device was attached to a package base and coated with organic silicon rubber on the edge to eliminate the interference of the reflected SAW. Finally, the IDTs were connected to the pins of the base via wire bonding.

### 2.2. Preparation of Sensitive Materials

**Figure 1:**
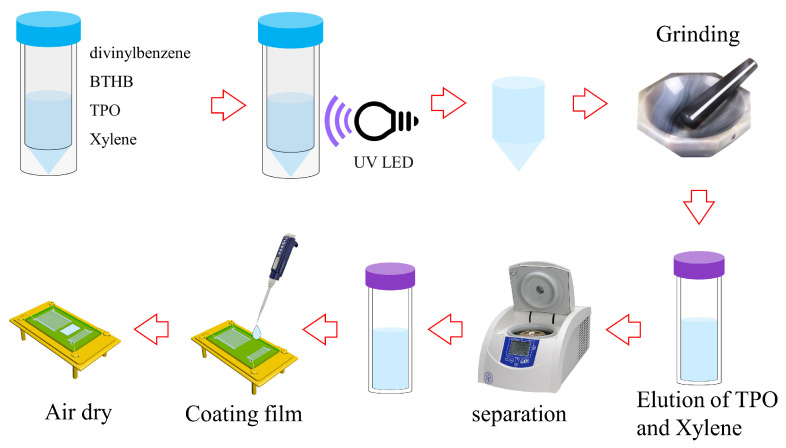
Preparation process of sensitive materials and the SAW sensor.

Information about the reagents used in this work is as follows. DMMP (98%) and xylene (analytic reagent) were from Aladdin (Aladdin Biochemical Technology Co., Ltd. Shanghai, China), and DVB (55%) and diphenyl (2,4,6-trimethylbenzoyl) phosphine oxide (TPO) were from Maclin (Macklin Biochemical Technology Co., Ltd., Shanghai, China). 2-Allylhexafluoroisopropanol (98%) was from Yuanye (Yuanye Bio-Technology Co., Ltd., Shanghai, China). It is crucial to note that the use of DVB without a stabilizer (p-tert-butylcatechol) is essential for obtaining a high-quality polymer, so the stabilizer must be removed from the DVB before conducting the experiment. The employed light source was an ultraviolet LED with a power of 1 W and a radiation wavelength of 365 nm. In a nitrogen-filled glove box, 0.107 mL of BTHB, 0.565 mL of DVB, 8.5 mL of xylene, and 5.8 mg of TPO were added to the centrifuge tube. After sealing the centrifuge tube, it was irradiated with the ultraviolet LED for 24 h to initiate the polymerization reaction, resulting in the formation of a bulk polymer material. The polymer was then ground into a slurry in a grinding bowl. The slurry was transferred to the centrifuge tube and 30 mL of ethanol was added to dissolve the xylene and TPO from the polymer. The centrifuge tube was placed in the centrifuge and centrifuged at 6000 RPM for 5 min to separate the polymer. This process was repeated three times to ensure the removal of xylene and TPO from the polymer. Subsequently, the polymer slurry was poured into the evaporation dish and dried at room temperature for 12 h to yield a white solid powder polymer. Solid polymer of 270 mg and 30 mL ethanol were added to the centrifuge tube and dispersed on the ultrasonic machine for 10 min to obtain a dispersion solution with a concentration of 9 mg/mL. Finally, a gas sensor was prepared by dropping 5 μL of the dispersion onto a SAW device using a microsyringe, followed by drying. The detailed preparation process is depicted in Figure 1.

According to the formulation outlined in Table 1, five samples with different HFIP contents were prepared to investigate the effect of HFIP content in sensitive materials on the sensor performance.

### 2.3. Material Characterization

The scanning electron microscope (SEM) and energy dispersive spectrometer (EDS) device was a Regulus 8100 (Hitachi, Hitachinaka, Japan). The infrared absorption spectra were obtained using a Shimadzu UV-3600 UV-VIS-NIR machine (Shimadzu, Kyoto, Japan). The surface area of the polymer was measured using Micromeritics ASAP2020 equipment (Micromeritics, Norcross, GA, USA). The thermal stability of the material was assessed using a thermogravimetric analysis system (TGA, METTLER TOLEDO, Zurich, Switzerland). The network analyzer was an ADVANTEST R3765CG (Advantest, Tokyo, Japan).

### 2.4. Sensor Measurement

The gas sensor was evaluated using a laboratory-developed test system. A compact fan and a heater were incorporated within a 27 L chamber. The liquid DMMP was gradually injected into the heater, undergoing evaporation and dispersion to form a homogeneous gas. The vapor concentration of DMMP was calculated based on the density, drop volume, and molecular weight of DMMP. A network analyzer was employed to examine the S_21_ curve of the sensor. The S_21_ is defined as S_21_ = −20lg(v_2_/v_1_), here v_1_ is the signal voltage at the input port of the SAW device, and v_2_ is the signal voltage at the output port. The S_21_, also known as insertion loss, is the energy loss of electromagnetic waves passing through the SAW device. As the sensitive material absorbs the gas, the mass accumulates, resulting in a shift in the S_21_ curve of the sensor. The maximum value derived from the S_21_ curve serves as the characteristic signal of gas concentration. Additional details regarding the test system are provided in the Supporting Information, as illustrated in Figures S1 and S2.

### 2.5. Calculation of Materials

The molecular modeling software Avogadro 1.2.0 was utilized for establishing the molecular model, while the quantum chemistry software ORCA 5.0.2 was employed to investigate intermolecular forces. Initially, the r2SCAN-3c functional was applied to optimize the molecular structure [25]. Subsequently, Multiwfn was used to calculate the surface electrostatic potential (ESP) of the molecule and visually display its active site [26,27]. For determining the binding energy E_ads_, high-accuracy density functional ωB97M-V and def2-TZVP basis sets were utilized [28].

The porous materials primarily consist of benzene rings, carbon chains, and HFIP functional groups. Benzene rings and carbon chains form the framework of this porous materials, while HFIP functional groups distribute on its surface. The binding energy was calculated using the methylation model (HFIP functional group and benzene ring). Adsorbed molecules included various compounds encountered in everyday life. By calculating the binding energy between functional groups and gas molecules, the contribution of functional groups to adsorption was investigated.

## 3. Results

### 3.1. Material Characterization

**Figure 2:**
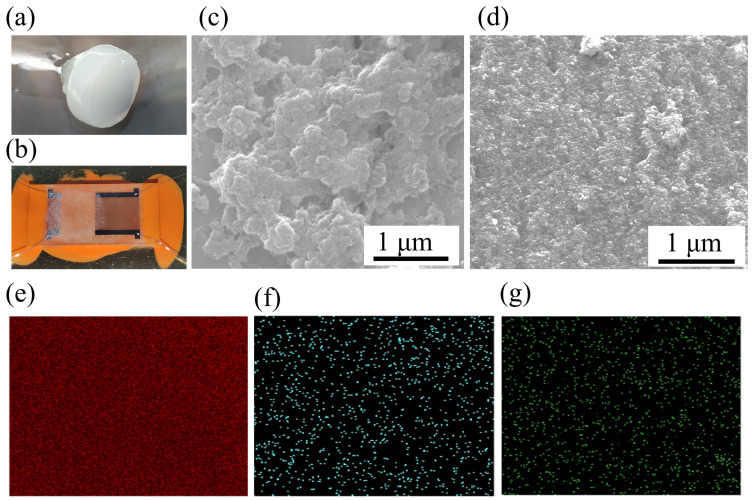
(**a**) Physical diagram of the bulk polymer. (**b**) The SAW gas sensor coated with sensitive materials. (**c**) Porous structure of bulk polymer. (**d**) SEM image of sensitive materials. EDS mapping of carbon (**e**), fluorine (**f**), and oxygen (**g**).

The initial polymer appears as a translucent mass, as shown in Figure 2a. After the removal of xylene from the bulk material by drying, numerous pores on the material could be observed via SEM, as illustrated in Figure 2c. Figure 2b shows the SAW gas sensor coated with a visible white sensitive film. SEM revealed that the sensitive film exhibited a rough granular morphology, as demonstrated in Figure 2d. The porous structure of this material facilitated easy grinding, enabling the production of particles with an approximate diameter of 1 μm. EDS was employed to examine the surface distribution of fluorocarbon and oxygen within the sensitive film, as shown in Figure 2e,f, respectively.

**Figure 3:**
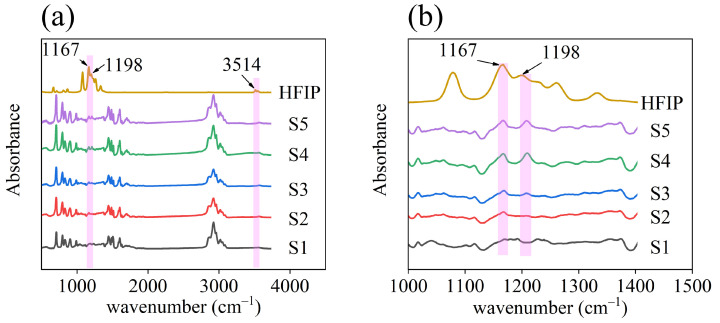
(**a**) Infrared absorption spectra of materials. (**b**) Fine structure of infrared absorption spectrum.

The chemical properties of the materials were investigated using infrared absorption spectroscopy. The infrared absorption spectra of the five samples and liquid hexafluoroisopropanol (used as a reference) were obtained, and the results are presented in Figure 3a. In the infrared absorption spectra of the HFIP porous materials, the peak at 3514 cm^−1^ corresponds to the characteristic peak of O-H stretching vibrations. The peaks at 1167 cm^−1^ and 1198 cm^−1^ correspond to the characteristic peaks of C-F stretching vibrations within the HFIP group, respectively. Figure 3b shows the fine structure of the infrared absorption spectra of the five samples. It can be discerned that as the amount of BTHB in the recipe increases, the intensity of the characteristic peak of HFIP in the five samples becomes increasingly prominent, suggesting an increase in the content of HFIP functional groups within the porous materials.

**Figure 4:**
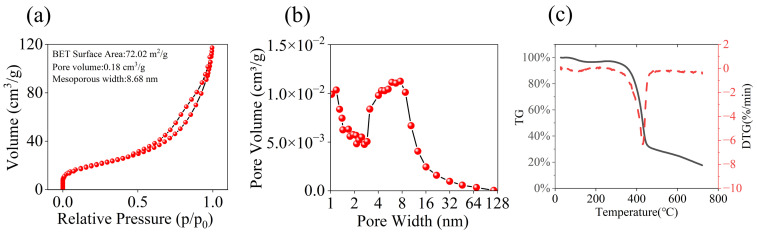
(**a**) Adsorption and desorption isotherm of sample S4. (**b**) The pore width of the S4 sample. (**c**) TG curve of S4 sample.

The test results of the sensor demonstrated that the sample S4 exhibited a higher response, prompting further investigation into its pore characteristics and thermal stability. As shown in Figure 4a, the nitrogen adsorption–desorption isotherm confirmed the synthesized material to be a type IVA mesoporous substance [29]. The BET surface area of the polymer was determined to be 72.02 m^2^/g, with a pore volume of 0.18 cm^3^/g and an average mesoporous width of 8.68 nm. The pore size distribution analysis depicted in Figure 4b revealed a predominant range of mesoporous widths between 3 and 10 nm. Additionally, the thermogravimetric (TG) curve presented in Figure 4c indicates a slight mass loss around 100 °C, which could be attributed to desorption of water vapor from the material’s surface. Significant mass loss started from 350 °C, which was due to the thermal decomposition of the polymer material. Combined with infrared absorption spectra and thermogravimetric curves, it is proved that HFIP groups are covalently bonded to the PAFs.

### 3.2. Sensor Performance Measurement and Optimization

**Figure 5:**
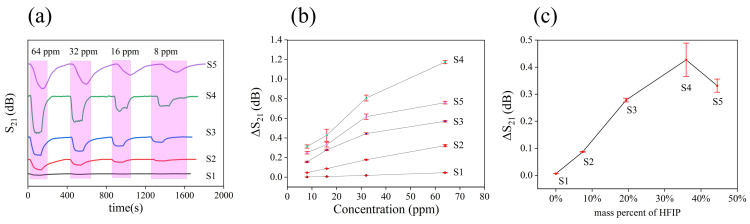
(**a**) Dynamic response curves of SAW sensors corresponding to five formulations. (**b**) The relationship between concentration of DMMP and response of SAW sensors. (**c**) The effect of HFIP mass percentage on response of sensor; DMMP concentration is 16 ppm.

The dynamic response curves of the five samples to different DMMP concentrations are depicted in Figure 5a. The relationships between concentration of DMMP and response of samples are illustrated in Figure 5b. The red line is the error bar, which represents the robustness of the sensor signal. Among the samples, S4 exhibited the highest sensitivity within the range of 8 ppm to 64 ppm. Figure 5c demonstrates the correlation between the mass percentage of HFIP content and the sensor response (at 16 ppm DMMP). As the HFIP mass percentage increased, the sensor response first increased and then decreased. This decrease occurred when the HFIP mass percentage reached 44%. As the HFIP groups content in the material increases, the sensitive material can adsorb more DMMP, so the response of the sensor is also improved. However, too many HFIP groups cause hydrogen bonds to form between the hydroxyl groups of HFIP. As a result, the response of sensor to DMMP is also reduced. To further support this mechanism, binding energy between HFIP functional groups was calculated. Compared with other samples, S1 sample was prepared by polymerization of DVB, and only a benzene ring and carbon chain existed in the material, without HFIP. Due to the very weak interaction between benzene and DMMP, the S1 sample showed a very low response to DMMP.

**Figure 6:**
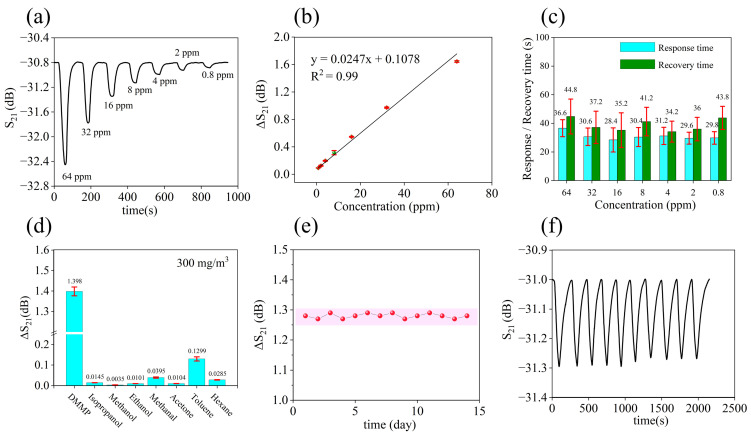
(**a**) Dynamic curve of the SAW gas sensor corresponding to S4 formulation. (**b**) The fitted curve between concentration and response. (**c**) Response time and recovery time of the SAW gas sensor. (**d**) Selectivity of the SAW gas sensor. (**e**) Aging performance of the SAW gas sensor. (**f**) Repeatability of the SAW gas sensor.

The sample S4 showed the highest response, so its performance was carefully evaluated. The response curve of sample S4 to different concentrations of DMMP is presented in Figure 6a, and the fitted curve between gas concentration and sensor response is illustrated in Figure 6b. The noise variance of the gas sensor is 4.9 × 10^−4^ dB, then the calculated LOD (limit of detection) of the gas sensor is about 0.11 ppm. Detailed noise data are shown in Figure S3. The response and recovery times of the sensor to DMMP are depicted in Figure 6c. Here, the response and recovery times were obtained using the t_90_ standard, that is, the time it takes for the response of sensor to reach 90% of its maximum response. The response time of the sensor at 0.8 ppm DMMP was 29.8 s, and the recovery time was 43.8 s. Within the tested concentration range, the average response and recovery times were 31.4 s and 38.9 s, respectively. For the prepared SAW sensors, response and recovery times within 1 min were achieved over the entire concentration range. Especially at low concentrations, rapid response can be maintained, which is beneficial for nerve agent sensors. Figure 6d displays the response value of the sensors to a variety of compounds with a concentration of 300 mg/m^3^, and the results suggest that the HFIP porous polymers exhibit excellent selectivity towards DMMP. Detailed data for selectivity are shown in Figure S4 and Table S1. The sensor has an obvious response to toluene, which is caused by the π–π interaction between the benzene ring in the sensitive material and toluene. This interaction leads to a high response of the sensitive material to toluene, to a certain extent. The responsiveness (represented by a green ball) of the sensor over a 14-day period at a DMMP concentration of 8 ppm was tested, as depicted in Figure 6e. The test results indicate that there was almost no attenuation after 14 days. In Figure 6f, the ten test cycles to 8 ppm DMMP demonstrate that the sensor has good repeatability. Overall, the SAW gas sensors coated with HFIP porous polymers show promising performance in detecting DMMP.

**Figure 7:**
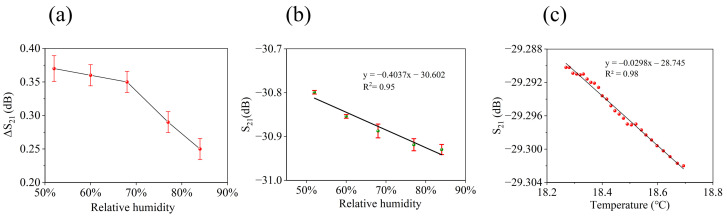
(**a**) The effect of humidity on the response of the gas sensor. (**b**) The effect of humidity on the baseline of the gas sensor. (**c**) The effect of temperature on the baseline of the gas sensor.

In practical application, it should be noted that humidity and temperature are important factors that affect the baseline and sensitivity of the SAW gas sensors. In Figure 5b, the response of sample S4 to 64 ppm DMMP is about 1.2 dB. In Figure 6b, the response of S4 is about 1.6 dB at the same concentration. This is due to the fact that the two tests were conducted in May and October, respectively. The ambient temperature and humidity in October are lower than in May, so the response of the sensor increases. The influence of temperature and humidity on the performance of the sensor was also studied in this work, as shown in Figure 7. The response of the sensor to 8 ppm DMMP under various humidity conditions is presented in Figure 7a, with detailed data provided in Figure S5. The responsiveness of the sensor gradually decreased as humidity levels rose, which can be attributed to the formation of hydrogen bonds between water and HFIP. This interaction reduces the availability of HFIP functional groups for adsorption of DMMP. The effect of humidity on the baseline signal of the sensor is demonstrated in Figure 7b. Increased humidity results in a lower baseline, suggesting that water molecules can be adsorbed by HFIP porous polymers. The relationship between humidity and the baseline signal is approximately linear, with an R^2^ value of 0.95 for the fitted curve. In clean air, the effect of temperature on the baseline signal is shown in Figure 7c. Figure S6 shows the system for testing the baseline and temperature of SAW sensors. The test results reveal a linear relationship between temperature and the baseline signal, with an R^2^ value of 0.98. The effect of temperature on the sensor baseline was tested on the fixture shown in Figure S6b, while the sensor performance was tested on the fixture shown in Figure S1d. Due to the difference in fixtures, there is a difference of 1.3 dB between the two test results. In conclusion, the test results above indicate that both temperature and humidity are significant factors influencing the baseline drift of the sensor, and this also suggests that compensating for temperature and humidity fluctuations is a viable approach to address the issue of baseline drift in SAW gas sensors.

**Figure 8:**
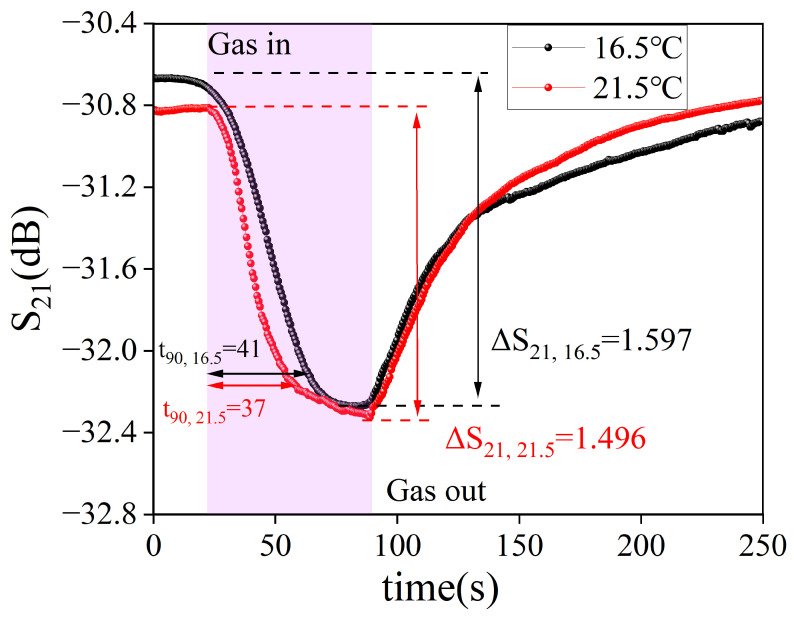
Response value and response time of the SAW gas sensor at different temperatures.

Temperature has a significant effect on the response value and speed of SAW gas sensors. To investigate this effect, the dynamic response curve of a SAW gas sensor coated with sample S4 was tested at 64 ppm DMMP. The dynamic response curves of the sensor at 16.5 °C and 21.5 °C were tested, respectively, and the results are shown in Figure 8. At 16.5 °C, the response value and response time of the sensor were 1.597 dB and 41 s. At 21.5 °C, the response value and response time of the sensor were 1.496 dB and 37 s. As the temperature increases, the response value and response time of the sensor decrease. This phenomenon is caused by the physical adsorption mechanism. With the increase in temperature, the adsorbed molecules can obtain a certain amount of energy from the exterior and leave the surface. It is this process that leads to a decrease in sensor response value and response time.

A comparison of our gas sensors with previous studies is presented in Table 2. Among them, HFIP–ionic gel [30] and HFIP–CNT [16] have also been reported as sensitive materials by our team. In comparison, the HFIP porous material exhibits notable advantages in terms of its simple preparation process.

## 4. Discussion

### 4.1. Sensing Mechanism

**Figure 9:**
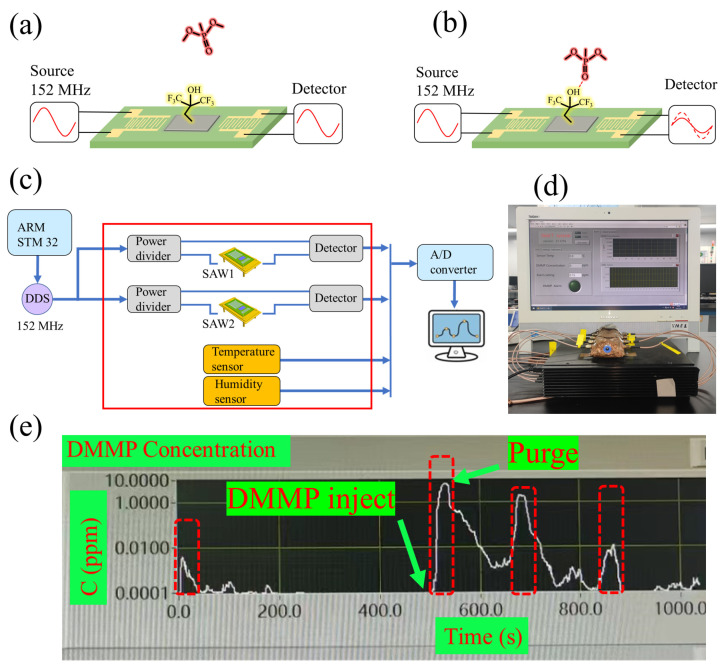
(**a**) The signal waveform of the detector when the sensitive material does not adsorb gas. (**b**) After the sensitive material adsorbs the gas, the signal waveform of the detector changes. (**c**) A schematic diagram of real-time detection equipment based on an SAW gas sensor. (**d**) A physical drawing of the real-time equipment. (**e**) Real-time response curve of the equipment to DMMP.

As shown in Figure 9a, the radio frequency source acts as a sine wave generator, while the detector measures the amplitude of the sine wave. A 152 MHz sine wave with amplitude v_1_ is introduced at the input port of the sensor to stimulate SAW on the piezoelectric material via the IDTs. The SAW propagates to the output port of the sensor and is converted by the IDTs into an electromagnetic wave signal with an amplitude of v_2_. The v_2_ is measured with the detector and the S_21_ is then calculated at the current frequency based on v_2_/v_1_. When DMMP molecules are adsorbed on the sensitive material, the total mass of the sensitive film increases, resulting in a decrease in SAW amplitude at the output port, as shown in Figure 9b. This phenomenon is often referred to as the mass loading effect [37,38]. The energy of the sine wave is proportional to v_1_^2^ and v_2_^2^, respectively, so the change in S_21_ represents the energy loss of SAW during propagation, which is caused by the mass loading effect. The greater the molecular mass of the gas molecule, or the smaller the mass of the sensitive film, the higher the sensitivity of the sensor. Therefore, carbon nanotubes, graphene, and PAFs are the preferred materials for increasing sensitivity.

Based on this mechanism, real-time DMMP detecting equipment was designed, as shown in Figure 9c. A sine wave generator (AD9910), driven by the ARM STM32 microcontroller, generates a 152 MHz sine wave signal. The sine wave is divided into two channels by the power divider; one channel is the reference signal, the other channel passes through the SAW1 sensor, and the amplitude difference of the two signals is tested by the detector (AD8302). In addition, temperature sensors and humidity sensors are used to obtain the temperature of the SAW device and the ambient humidity. The signals of all sensors are read by the A/D converter and output after further data processing by the computer. SAW1 is a sensor coated with HFIP porous polymers to detect nerve agents, and SAW2 is a sensor coated with polyepichlorohydrin to detect mustard gas. Figure 9d shows a physical drawing of the real-time equipment. Inside the black box is the circuit system, and the SAW sensor array on the box is wrapped in yellow copper foil. The detailed structure of this equipment is shown in Figure S7. A computer was used to process and display the test data in real time. When DMMP vapor was injected into the SAW sensor, the system detects an increase in DMMP concentration, which decreases to 0 after purging the sensor. This test was conducted four times (marked by the red dotted box), and the real-time concentration curve is shown in Figure 9e.

**Figure 10:**
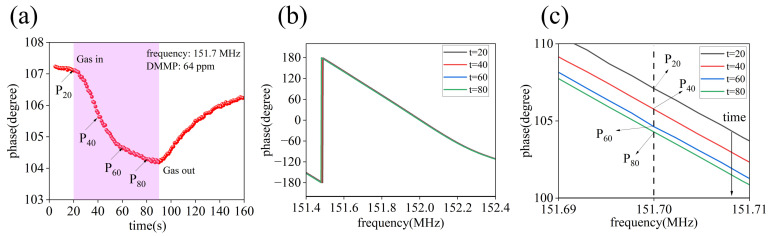
(**a**) Dynamic response curve of SAW sensor in phase mode. (**b**) The phase curve of the sensor at four moments. (**c**) Detailed phase curve.

In fact, after the SAW sensor adsorbs the gas, SAW delay also occurs. This delay is ultimately reflected in a change in the phase of the electromagnetic wave signal at the output port of the SAW device. Like the S_21_ curve, the phase curve is also a function of frequency. The phase characteristics of the SAW sensor were tested at 64 ppm DMMP, and the test results are shown in Figure 10. Here, the phase of an electromagnetic wave with a frequency of 151.7 MHz is extracted from the phase curve as a concentration characteristic signal. The dynamic response curve of the sensor to 64 ppm DMMP is shown in Figure 10a. When DMMP was injected into the chamber, the phase of the sensor gradually reduced from 107° to 104°. Figure 10b shows the phase curve of the SAW sensor at four moments, and Figure 10c shows the detailed curve near 151.7 MHz. The results confirm that the phase curve of the sensor moves to the left when the sensitive film adsorbs DMMP gas.

### 4.2. Adsorption Mechanism

**Figure 11:**
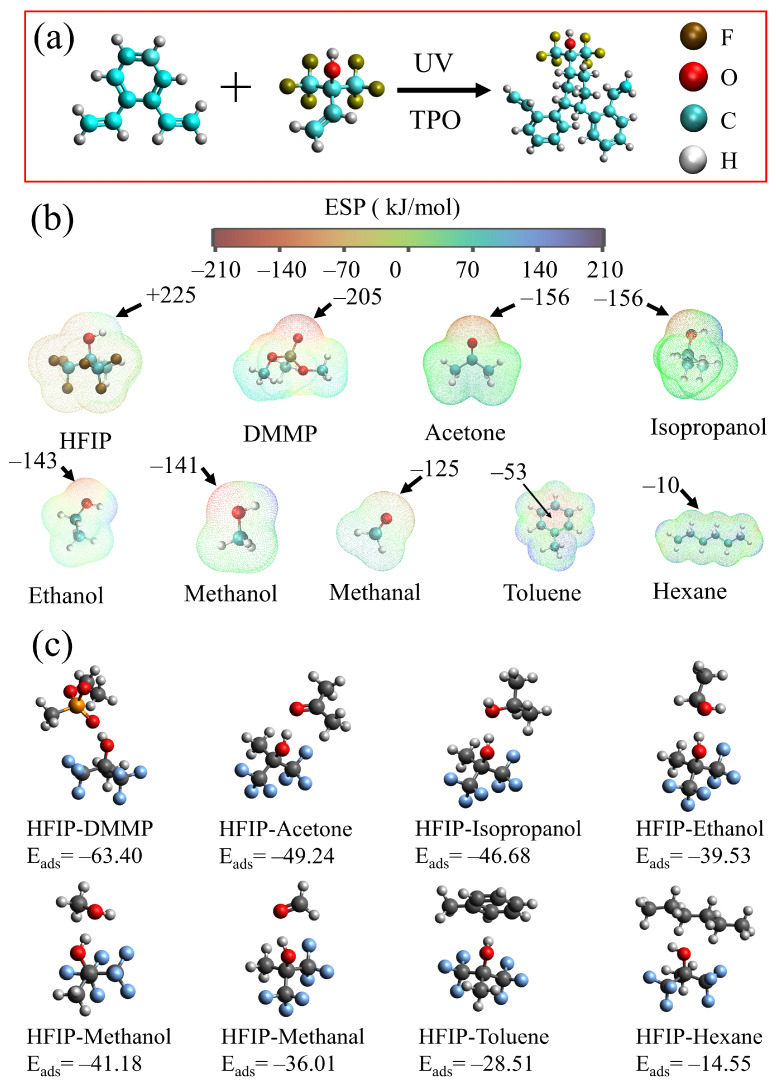
(**a**) The polymerization of DVB and BTHB. (**b**) ESP of gas molecules. (**c**) Binding energy of HFIP and molecules.

The BTHB and DVB compounds both contain vinyl functional groups, and under ultraviolet irradiation, they undergo slow polymerization at room temperature. The reaction process is illustrated in Figure 11a. The inclusion of xylene in the recipe results in the formation of numerous pores within the polymer structure. These porous frameworks facilitate the exposure of HFIP groups and enhance the diffusion of DMMP molecules into the polymer matrix.

Figure 11b depicts the ESP of various molecules. The H atom on the hydroxyl of HFIP possesses a positive ESP (+225 kJ/mol), whereas the O atom on the P=O group of DMMP has a negative ESP (−205 kJ/mol). Based on the complementary relationship between their ESPs, it can be inferred that the H atom of hydroxyl group tends to form a hydrogen bond with the O atom of DMMP [39]. Here, the potential binding sites between the two molecules are preliminarily determined and the strength of binding is reflected by binding energy E_ads_. Binding energy E_ads_ is defined as the difference between the total energy of two molecules before and after binding, and this difference is usually negative. The more negative this energy is, the more energy the two molecules release before and after binding, and the more stable the binding between molecules is. The calculated E_ads_ between DMMP and HFIP is −63.40 kJ/mol, which indicates a strong hydrogen bond between DMMP and HFIP [40]. Figure 11b,c also illustrate the ESP of seven common compounds and their binding energy with HFIP. In acetone, isopropanol, methanol, ethanol, and methanal, the O atom exhibits a significantly negative ESP, indicating the potential formation of hydrogen bonds with HFIP. Regarding toluene, a negative ESP region is situated within the benzene, characterized by a binding energy of −33.24 kJ/mol between the active site and HFIP. The molecular surface of hexane exhibits distinct regions with a notable negative ESP, reaching an extreme value of −10 kJ/mol. The binding energy between hexane and HFIP is −14.55 kJ/mol. In comparison, these seven molecules exhibit higher binding energies with HFIP than DMMP does, suggesting that the hydrogen bonds formed between these seven molecules and HFIP were weak. This result indicates that when the HFIP polymer is placed in a mixed atmosphere of eight molecules, HFIP will preferentially adsorb DMMP. This provides a theoretical rationale for the preferential adsorption of DMMP on HFIP. Detailed calculation data can be found in Table S2 of Supporting Information. It should be noted that porous materials containing benzene can generate π–π interactions with toluene; therefore, it is reasonable for sensors to show response to toluene.

In Figure S8, the ESP of 1,2-dipropylbenzene is presented. The findings reveal that the center of the benzene possesses a negative ESP, while the H atom on the benzene exhibits a positive ESP. The calculation outcomes of binding energies between the model and diverse molecules are delineated in Figure S9 and Table S3. Compared with HFIP, the interaction between the benzene of 1,2-dipropyl benzene and gas molecules is notably weaker.

### 4.3. Interaction between HFIPs

**Figure 12:**
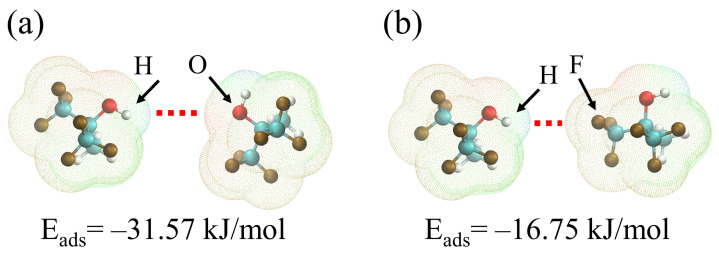
Binding energy between HFIPs. (**a**) The bond between the H atom and the O atom. (**b**) The bond between the H atom and the F atom.

During the investigation, it was observed that the sensor response did not proportionally increase with the content of HFIP in the material, as depicted in Figure 5c. For the HFIP group, the O and F atoms exhibit negative ESP, while the H atoms in the hydroxyl group display positive ESP. Hydrogen bonds (indicated by the red dashed line) tend to form between the two atoms, as shown in Figure 12. The binding energies between the H atom of the hydroxyl group and O and F were calculated separately. The intermolecular bonding energies between HFIPs are −31.57 kJ/mol and −16.75 kJ/mol, respectively. This indicates that the bonding between hydroxyl groups is more robust than that between hydroxyl groups and F atoms, facilitating the formation of stable hydrogen bonds between the hydroxyl groups of HFIP. As the content of HFIP in the material increases, the rate of hydrogen bonds forming between HFIPs amplifies, thereby diminishing the quantity of hydroxyl groups capable of adsorbing DMMP. The presence of this mechanism limits the LOD of flexible materials such as HFIP-modified polydimethylsiloxane, because the molecular chains of polydimethylsiloxane can move, which allows hydrogen bonds to form between HFIP functional groups in the material. To further improve sensor response and prevent bonding between HFIPs, the use of porous frame materials is a potential strategy.

## 5. Conclusions

In summary, this work has developed a novel HFIP-modified porous polymer that can be used to detect nerve agents and DMMP. The preparation of porous polymers using UV-induced polymerization demonstrted the simplicity of the process, which is necessary for mass production of nerve agent sensors. The pores provide a gas diffusion channel, which enables the sensor to exhibit high sensitivity and fast response speed. The SAW sensor has a response/recovery time of 29.8/43.8 s to 0.8 ppm DMMP and an LOD of approximately 0.11 ppm. It was found that the baseline of the sensor has a linear relationship with temperature and humidity. As the humidity increases, the response of the sensor decreases. As the temperature increases, the sensitivity and response time of the sensor decrease. Based on the amlitude detection method, a DMMP real-time detection device has been designed. The interaction between functional groups and gas molecules w studied, and the interaction between HFIPs should be considered as an important factor limiting the sensor response. After comprehensive experimental and theoretical research, the developed SAW gas sensor has great potential in nerve agent detection.

## Figures and Tables

**Table 1:** Formulation for preparing sensitive materials.

Sample	BTHB (mL)	DVB (mL)	Xylene (mL)	TPO (g)	HFIP Mass Ratio
S1	0	0.565	8.5	0.0058	0%
S2	0.015				7%
S3	0.046				19%
S4	0.107				36%
S5	0.153				44%

**Table 2:** Sensor performance comparison.

Materials	Device	Key Process	Response Time/Recovery Time	Detection Concentration (ppm)	Ref.
HFIP—ionic gel	SAW sensor	Photoinduced polymerization and patterning	23 s/58 s	0.2	[30]
HFIP—CNT	SAW sensor	Surface modified HFIP and self-assembly process	3 s/50 s	0.1	[16]
HFIP—3D carbon	Resistor	Electrospinning machine and in-situ CVD technique	2.4 s/4.7 s	0.1	[31]
HFIP—Co_3_O_4_/CuO	Resistor	Electrospinning machine and surface modified HFIP	7.3 s/5.2 s	0.5	[32]
HFIP—WO_3_/rGO	Resistor	Electrospinning machine and surface modified HFIP	23 s/25 s	0.1	[17]
rGO—GNC	Resistor	Hydrothermal method and drop-casting	105 s/150 s	10	[33]
Poly-3-hexylthiophene	SAW sensor	Spray-coating technology and light activation of the sensitive material	100 s/240 s	0.133	[34]
PMFOS	SAW sensor	Chemical synthesis of PMFOS and thermal evaporation coating	100 s/480 s	0.013	[35]
3D hybrid Ni-CNT	Resistor	Electrospinning machine and and drop-casting	11 s/12 s	0.1	[36]
HFIP porous polymers	SAW sensor	Photoinduced polymerization and drop-casting	29.8 s/43.8 s	0.8	this work

## Data Availability

The data supporting the findings of this study are available from the corresponding author upon reasonable request.

## Supplementary Materials

The following supporting information can be downloaded at: https://www.mdpi.com/article/10.3390/nano14010089/s1, Figure S1. (a) The structure diagram of the test system. (b) The overall appearance of the test system. (c) Heater for producing DMMP vapor. (d) A SAW gas sensor and its fixture. Figure S2. (a) The S_21_ curve of the sensor at different times. (b) The detailed structure of the S_21_ curve. (c) The dynamic response curve of the sensor. Figure S3. Noise characteristics of SAW gas sensors. Figure S4. Selectivity of gas sensors at 300 mg/m^3^. Figure S5. Response curve of the sensor to DMMP at different humidity. Figure S6. (a) The system for testing the baseline and temperature of SAW sensors. (b) Refrigeration system for cooling the SAW sensor. Figure S7. (a) The circuit system of real-time equipment in black box. (b) The SAW gas sensor and Sensor array. Figure S8. ESP of 1,2-dipropylbenzene. Figure S9. Binding energy and binding site between 1,2-dipropylbenzene and molecules. Table S1. Mass concentration (300 mg/m^3^) corresponding volume concentration (ppm). Table S2. Binding energy of HFIP and molecules. Table S3. Binding energy between 1,2-dipropylbenzene and molecules.
