# Supplementary material for: A Nanoporous Polymer Modified with Hexafluoroisopropanol to Detect Dimethyl Methylphosphonate
Source: Nanomaterials (Basel). 2023 Dec 28;14(1):89. doi: 10.3390/nano14010089 (PMC10781009; doi:10.3390/nano14010089)
Supplement: Supplementary file 1 [file nanomaterials-14-00089-s001.zip › nanomaterials-2748264-supplementary.pdf]

Supporting Information

A Nanoporous Polymer Modified with Hexafluoroisopropanol to Detect Dimethyl Methylphosphonate

Xuming Wang [1], Xin Li [1,*], Qiang Wu [1], Yubin Yuan [1], Weihua Liu [1], Chuanyu Han [1] and Xiaoli Wang [2]

1 Department of Microelectronics, Xi'an Jiaotong University, Xi'an 710049, China

2 School of Physics, Xi'an Jiaotong University, Xi'an 710049, China

* Correspondence: lx@mail.xjtu.edu.cn

The performance of the SAW sensor was evaluated using a laboratory test system. Figure S1a illustrates the structural diagram of the test system, while figure S1b depicts its physical arrangement. The test chamber, multimeter (Keithley2000) and network analyzer are arranged from top to bottom. A Pt100 thermistor is affixed to the fixture as a temperature sensor, which is connected to a multimeter. By connecting the network analyzer and multimeter to a computer, we obtained both the response curve and temperature readings of the sensor.

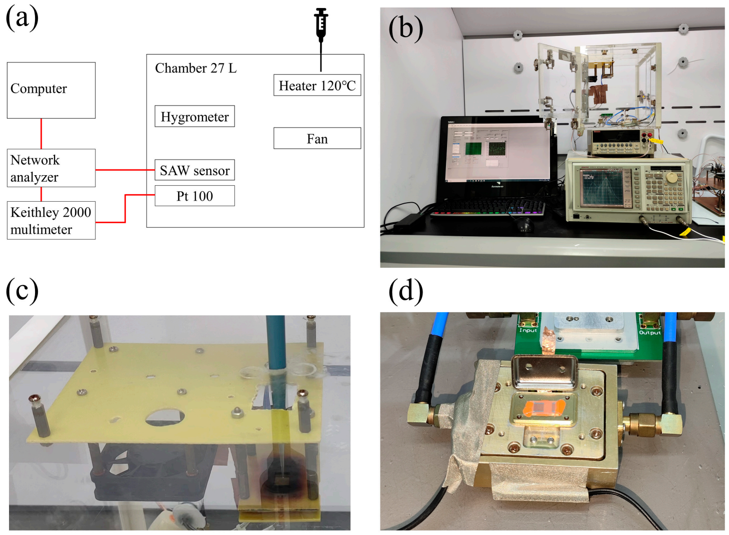

Figure S1. (a) The structure diagram of the test system. (b) The overall appearance of the test system. (c) Heater for producing DMMP vapor. (d) A SAW gas sensor and its fixture.

The chamber is constructed from acrylic, with internal dimensions of 30 cm in length, width, and height, resulting in a volume of 27 L, as shown in figure S1b. Positioned at the top of the chamber are a small heater operating at 120℃ and a fan for air circulation, as shown in figure S1c. A fixture designed for accommodating the SAW sensor is installed at the bottom of the chamber, as shown in figure S1d. Additionally, there exists an aperture on top with a lid facing the heater. The SAW sensor should be mounted onto the fixture before securely closing the chamber door. To generate gaseous DMMP, a specific quantity of liquid DMMP is carefully dispensed onto the heated

surface using a micro syringe. Subsequently, activating the fan ensures uniform distribution of evaporated DMMP gas throughout the chamber within approximately 3 seconds. The concentration level of DMMP vapor can be controlled by adjusting injected liquid volume and evaluated utilizing equation (1).

$$c(\mathrm{ppm}) = (\mathrm{V_{DMMP}} \times \rho_{DMMP} \div \mathrm{M_{DMMP}}) \times 0.907 \times 10^6 \quad (1)$$

where $\mathrm{V_{DMMP}}$, ρ_{DMMP} and $\mathrm{M_{DMMP}}$ were the volume, density and molar mass of DMMP, respectively. The test system is placed in a fume hood. After the test process is complete, the chamber was opened and purged with air until the sensor returned to its initial state.

The system is capable of investigating the dynamic response and selectivity of the gas sensor, as well as assessing the impact of humidity and temperature on its performance.

The principle of testing SAW sensors using a network analyzer is presented below. When gas molecules are adsorbed by the sensitive material of the SAW sensor, there is a significant alteration in the S_{21} curve. A network analyzer is employed to measure the S_{21} curve of the SAW gas sensor at various time intervals. An exemplary test curve is depicted in figure S2a, where different colors represent distinct time points on the S_{21} curve. The maximum P_t (t denoting time) is extracted from the S_{21} curve as an output signal of the SAW sensor. As illustrated in figure S2b, P_{30} denotes the peak value of the S_{21} curve observed at 30s for this sensor. These output signals collectively formulate a dynamic response curve for the sensor, as demonstrated in figure S2c.

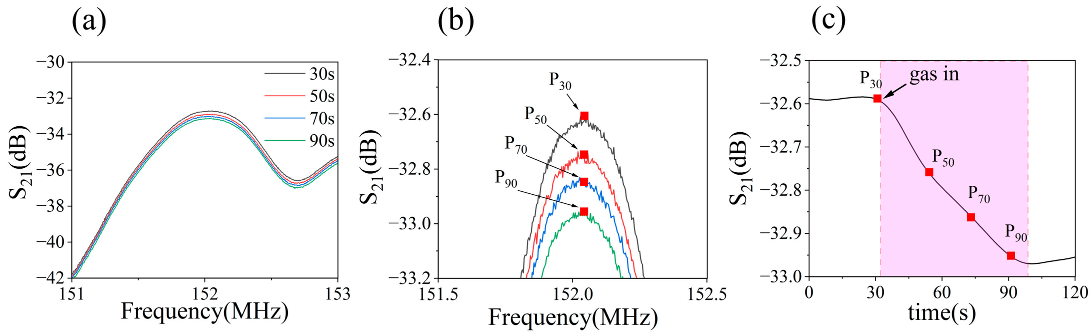

Figure S2. (a) The S_{21} curve of the sensor at different times. (b)The detailed structure of the S_{21} curve. (c) The dynamic response curve of the sensor.

The SAW sensor coated with S4 sample was tested in clean air for 160 s to obtain its noise variance, and the noise distribution of the sensor is shown in figure S3. The results show that the average S_{21} of the sensor within 160 s is −30.8275 dB, the S_{21} presents a normal distribution, and the noise variance is 4.9×10^{-4} dB.

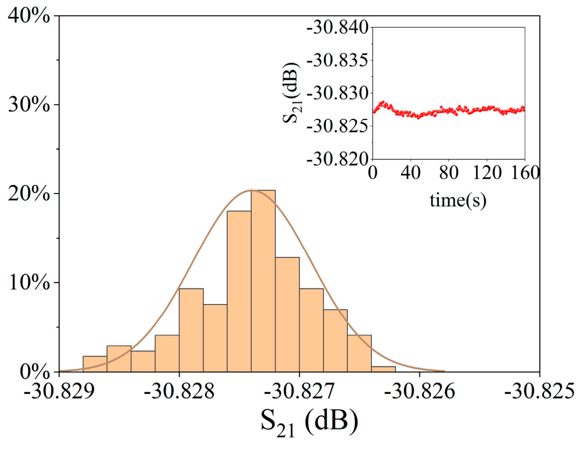

Figure S3. Noise characteristics of SAW gas sensors.

The selectivity of the gas sensor is a crucial parameter, and 8 gases with a concentration of 300 mg/m^3 were chosen to evaluate the selectivity of sensor. Figure S4 illustrates the dynamic response curve of the sensor towards different gases

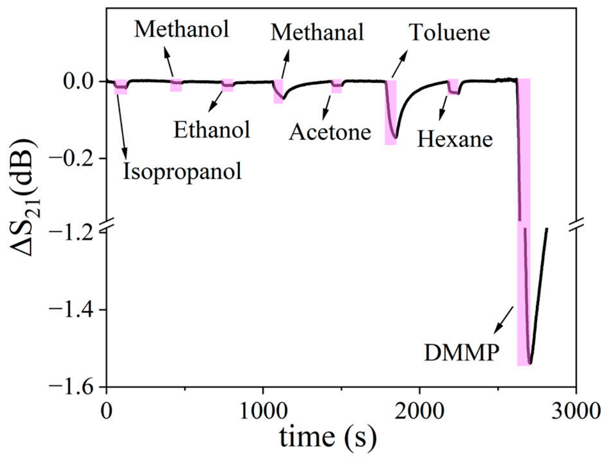

Figure S4. Selectivity of gas sensors at 300 mg/m^3.

Due to the mass loading effects that underlie the operating principle of SAW gas sensors, variations in molecular mass can lead to disparate responses even at identical volume concentrations. This phenomenon may potentially mislead researchers when evaluating sensor selectivity. Hence, we employ mass concentration as the criterion for assessing selectivity, and provide a corresponding relationship between mass concentration and volume concentration in Table S1 for ease of calculation.

Table S1. Mass concentration (300 mg/m^3) corresponding volume concentration (ppm).

Compounds	Mass concentration (mg/m^3)	Volume concentration (ppm)
DMMP	300	54
Isopropanol	300	112
Methanol	300	209
Ethanol	300	146
Methanal	300	224
Acetone	300	116
Xylene	300	73
Hexane	300	78

The response curve of the sensor under varying humidity are illustrated in figure S5. A two-step methodology was employed to investigate the effect of humidity on the response of the sensor. Initially, a specific volume of water was introduced into the evaporator within the chamber to establish a stable humidity level. Subsequently, liquid DMMP was injected into the evaporator to generate DMMP gas and evaluate the corresponding sensor response at this particular humidity level.

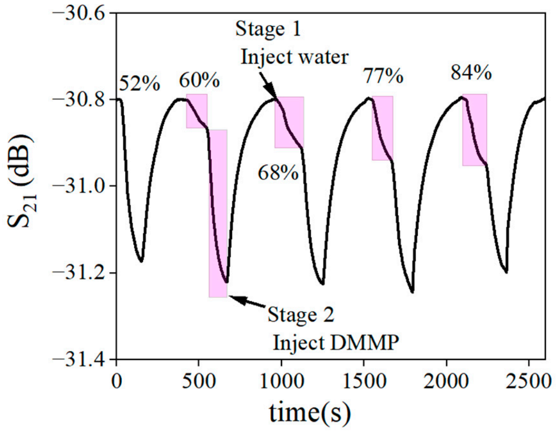

Figure S5. Response curve of the sensor to DMMP at different humidity.

In order to study the effect of temperature on the baseline of the SAW gas sensor, a test system was set up, as shown in figure S6a. The Keithley 2000 and Pt100 thermistor are used to test the temperature of the SAW gas sensor. While obtaining the temperature of the SAW sensor, the S_{21} curve of the sensor is measured using the network analyzer. Figure S6b shows the refrigeration system. Here, a semiconductor chilling plate with a maximum power of 60 w is used to reduce the temperature of the SAW sensor, and a temperature controller is used to control the temperature of the semiconductor chilling plate. The fixture of the SAW device and the Pt100 thermistor are placed close to the semiconductor chilling plate.

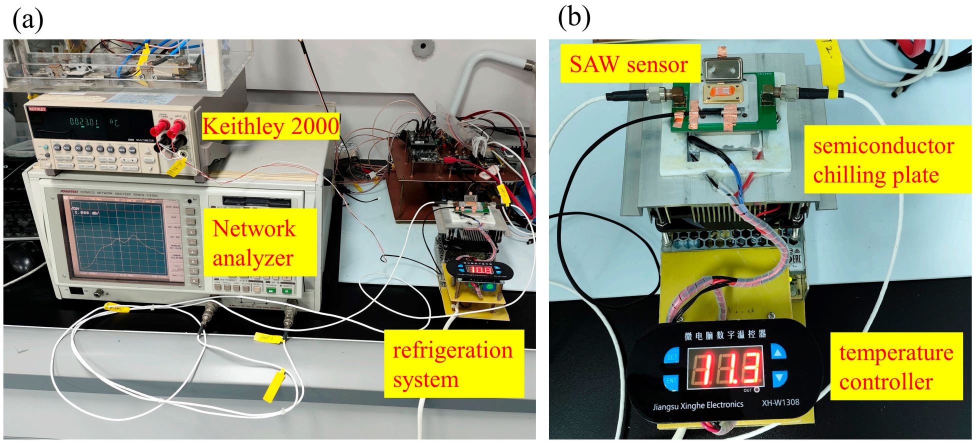

Figure S6. (a) The system for testing the baseline and temperature of SAW sensors. (b) Refrigeration system for cooling the SAW sensor.

Figure S7a shows the designed real-time detection equipment. Sinusoidal radio frequency signal source (DDS), STM32 MCU, power divider, detector and temperature and humidity module are installed in the black box. The sensor array is wrapped in copper foil to reduce interference from external electrical signals. Figure S7b shows one of the SAW gas sensors, which is mounted in a small aluminum chamber.

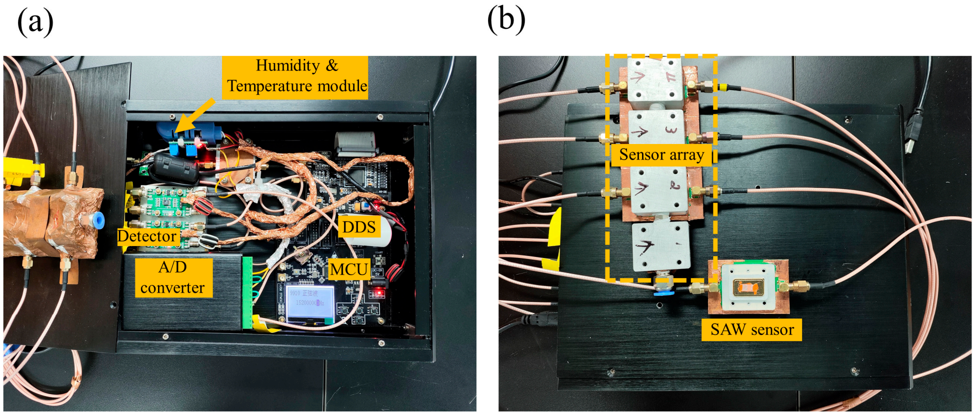

Figure S7. (a) The circuit system of real-time equipment in black box. (b) The SAW gas sensor and Sensor array.

Table S2. Binding energy of HFIP and molecules.

Adsorbed molecule	Total energy (kJ/mol)	HFIP energy (kJ/mol)	Energy of adsorbed molecule (kJ/mol)	Binding energy (kJ/mol)
DMMP	−3980716.99	−2177308.68	−1803344.89	−63.4
Isopropanol	−2687582.65	−2177309.46	−510226.5	−46.68
Methanol	−2481170.26	−2177315.5	−303813.55	−41.18
Ethanol	−2584368.09	−2177309.65	−407018.9	−39.53
Methanal	−2477987.6	−2177309.63	−300641.93	−36.01
Acetone	−2684454.26	−2177309.35	−507095.65	−49.24
Toluene	−2888907.81	−2176298.33	−712580.91	−28.51
Hexane	−2799591.86	−2177311.19	−622266.14	−14.55

The ESP of the 1, 2-dipropylbenzene molecule is shown in figure S8. The central position of the benzene ring exhibits a negative ESP, which facilitates an electrostatic attraction with the methyl group of the DMMP. Meanwhile, the hydrogen atom located at the edge of the benzene ring displays a positive ESP, enabling it to attract with the oxygen atom in DMMP.

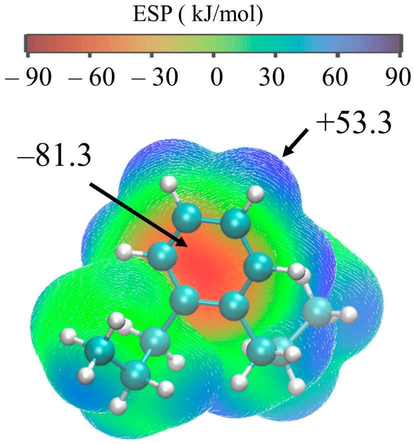

Figure S8. ESP of 1,2-dipropylbenzene.

The binding sites and energy between 1,2-dipropylbenzene and eight molecules are illustrated in figure S9. Table S3 list the binding energy of 1,2-dipropylbenzene and molecules.

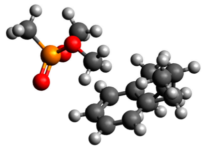

Dipropylbenzene
-DMMP
E_{ads}= –27.37

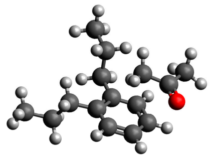

Dipropylbenzene
- Acetone
E_{ads}= –21.8

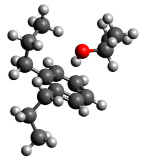

Dipropylbenzene
- Isopropanol
E_{ads}= –28.38

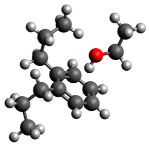

Dipropylbenzene
- Ethanol
E_{ads}= –28.61

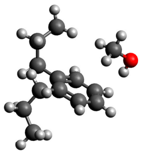

Dipropylbenzene
- Methanol
E_{ads}= –16.82

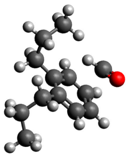

Dipropylbenzene
- Methanal
E_{ads}= –18.04

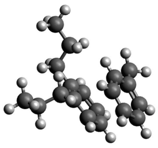

Dipropylbenzene
- Toluene
E_{ads}= –26.05

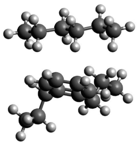

Dipropylbenzene
- Hexane
E_{ads}= –25.44

Figure S9. Binding energy and binding site between 1,2-dipropylbenzene and molecules.

Table S3. Binding energy between 1,2-dipropylbenzene and molecules.

Adsorbed molecule	Total energy (kJ/mol)	1,2-Dipropylbenzene energy (kJ/mol)	Energy of adsorbed molecules (kJ/mol)	Binding energy (kJ/mol)
DMMP	−3030835.65	−1228314.31	−1802493.96	−27.37
Acetone	−1735199.98	−1228317.62	−506860.53	−21.83
Toluene	−1940925.9	−1228317.82	−712582.03	−26.05
Methanol	−1532002.32	−1228313.98	−303671.53	−16.82
Methanal	−1528836.02	−1228315.68	−300502.29	−18.04
Ethanol	−1635172.99	−1228315.71	−406830.47	−26.81
Isopropanol	−1738334.93	−1228315.78	−509990.77	−28.38
Hexane	−1850319.94	−1228317.6	−621976.9	−25.44
